# Novel Preprocessing-Based Sequence for Comparative MR Cervical Lymph Node Segmentation

**DOI:** 10.3390/jcm14061802

**Published:** 2025-03-07

**Authors:** Elif Ayten Tarakçı, Metin Çeliker, Mehmet Birinci, Tuğba Yemiş, Oğuz Gül, Enes Faruk Oğuz, Merve Solak, Esat Kaba, Fatma Beyazal Çeliker, Zerrin Özergin Coşkun, Ahmet Alkan, Özlem Çelebi Erdivanlı

**Affiliations:** 1 Department of Otorhinolaryngology, Medicine Faculty, Recep Tayyip Erdoğan University, Rize 53000, Turkey; elifayten.tarakci@erdogan.edu.tr (E.A.T.); mehmet.birinci@erdogan.edu.tr (M.B.); tugba.yemis@erdogan.edu.tr (T.Y.); zerrin.coskun@erdogan.edu.tr (Z.Ö.C.); ozlem.erdivanli@erdogan.edu.tr (Ö.Ç.E.); 2 Department of Otorhinolaryngology, Akçaabat Haçkalı Baba State Hospital, Trabzon 61310, Turkey; drogzgl@gmail.com; 3 Department of Biomedical Device Technology, Hassa Vocational School, Hatay Mustafa Kemal University, Hatay 31000, Turkey; farukenes.oguz@mku.edu.tr; 4 Department of Radiolagy, Medicine Faculty, Recep Tayyip Erdoğan University, Rize 53000, Turkey; mervesolak2555@gmail.com (M.S.); esat.kaba@erdogan.edu.tr (E.K.); fatma.bceliker@erdogan.edu.tr (F.B.Ç.); 5 Department of Electrical and Electronics Engineering, Kahramanmaraş Sütçü İmam University, Kahramanmaraş 46000, Turkey; aalkan05@gmail.com

**Keywords:** cervical lymph node, magnetic resonance imaging, artificial intelligence, deep learning, segmentation

## Abstract

**Background and Objective**: This study aims to utilize deep learning methods for the automatic segmentation of cervical lymph nodes in magnetic resonance images (MRIs), enhancing the speed and accuracy of diagnosing pathological masses in the neck and improving patient treatment processes. **Materials and Methods**: This study included 1346 MRI slices from 64 patients undergoing cervical lymph node dissection, biopsy, and preoperative contrast-enhanced neck MRI. A preprocessing model was used to crop and highlight lymph nodes, along with a method for automatic re-cropping. Two datasets were created from the cropped images—one with augmentation and one without—divided into 90% training and 10% validation sets. After preprocessing, the ResNet-50 images in the DeepLabv3+ encoder block were automatically segmented. **Results**: According to the results of the validation set, the mean IoU values for the DWI, T2, T1, T1+C, and ADC sequences in the dataset without augmentation created for cervical lymph node segmentation were 0.89, 0.88, 0.81, 0.85, and 0.80, respectively. In the augmented dataset, the average IoU values for all sequences were 0.91, 0.89, 0.85, 0.88, and 0.84. The DWI sequence showed the highest performance in the datasets with and without augmentation. **Conclusions**: Our preprocessing-based deep learning architectures successfully segmented cervical lymph nodes with high accuracy. This study is the first to explore automatic segmentation of the cervical lymph nodes using comprehensive neck MRI sequences. The proposed model can streamline the detection process, reducing the need for radiology expertise. Additionally, it offers a promising alternative to manual segmentation in radiotherapy, potentially enhancing treatment effectiveness.

## 1. Introduction

A neck mass is a symptom that can affect all age groups and is a very worrying symptom for patients. In order to make a differential diagnosis of neck masses, it is necessary to have detailed information about the diagnosis of many diseases. The mass in the neck may be because of a simple infection, or it may be the first symptom of a malignancy originating from the head and neck region, or the distant metastasis of a malignancy in another part of the body. To diagnose a head and neck mass, doctors should perform a detailed physical examination, imaging, and biopsy [1,2].

The stage of head and neck malignancies at diagnosis predicts the prognosis and survival rate of patients. According to the 8th edition of the AJCC Cancer Staging Manual, the presence of only one lymph node metastasis leads to a higher tumour stage in most types of head and neck cancer. Therefore, the accurate identification and characterisation of lymph node metastases has critical prognostic importance [3]. Magnetic resonance imaging (MRI) of neck masses with suspected malignancy can differentiate the soft tissue spread of pathology and malignancy with high accuracy. Evaluation of MRI requires experience and expertise in radiology [4]. In recent years, deep learning techniques, which have found their place in every aspect of our lives, have revolutionised this process.

Deep learning is a special type of artificial neural network that resembles the human nervous system. Artificial neural networks comprise units connected by links. A connection spreads the activation it receives from one unit to another, and a numerical value that determines the strength of the connection expresses each connection activation. Given the current availability of big data, improved computing power with graphics processing units, and new algorithms for training deep neural networks, many limitations are now solved. These deep learning approaches are the most popular technique used in medical imaging, especially for image classification, lesion detection, and segmentation [5,6]. In this context, deep learning methods can segment cervical lymph nodes by analysing the data obtained from MRI images, making it possible to differentiate the lymph nodes from other structures in the neck and examine them. Such approaches can expediate the diagnosis of the disease and facilitate treatment strategies.

The aim of this study is to automatically segment neck lymph nodes, which are critical to evaluate before neck surgeries and are difficult and time consuming to detect on MRI. This will be accomplished by utilizing an innovative preprocessing method based on deep learning. Another aim of this study is to make an evaluation by comparing the performance of MR sequences in the segmentation of lymph nodes. For this purpose, we used all the images from five sequences (DWI, T2, T1+C, T1, and ADC).

## 2. Materials and Methods

### 2.1. Patient Selection (Data)

Patients who underwent neck dissection or incisional/excisional cervical lymph node biopsy and were diagnosed with head and neck squamous cell carcinoma or lymphoma in our hospital between January 2018 and December 2023 and had preoperative contrast-enhanced neck MRI and diffusion MRI of the neck were included in this study. We obtained all images from a 1.5 T MRI scanner. Exclusion criteria were patients whose images were of low quality and contained artifacts because of motion, breathing or foreign bodies, etc., or whose contrast timing was inappropriate, and patients who underwent 3 Tesla MRI in our hospital. After applying the exclusion criteria, we included 64 patients in this study from 112 patients with neck masses who had preoperative MRI.

### 2.2. MRI Protocol

A 32-channel head coil and a 1.5 T scanner (Siemens Magnetom Aera, Erlangen, Germany) were used to gain neck MR images. Before contrast, we obtained axial T1 images (TR = 400, TE = 8.6, FOV = 256 × 320, phase FOV = 100, slice thickness = 3 mm, NEX = 1) and axial T2 images (TR = 3820, TE = 96, FOV = 256 × 320, phase FOV = 100, slice thickness = 3 mm, NEX = 2). After 0.1 mmol/kg intravenous injection of gadolinium contrast agent (Meglumin Gadoterate, Dotarem, Guerbet, France), fat-suppressed T1 coronal images (TR = 471, TE = 12, FOV = 224 × 320, phase FOV = 100, slice thickness = 3 mm, NEX = 3) were gained in the axial plane. We performed DWI in the axial plane using an echo-planar single-shot spin-echo sequence with b values of 0, 1000, 2000, and 3000 s/mm^2^ (TR/TE 9000/79 ms). The workstation then generated ADC maps. All patients used pre-contrast T1, T2, DWI-b1000, ADC, and contrast-enhanced T1 (T1c)-weighted images. The workstation created all slices, including cervical lymph nodes, in the axial plane using five different sequences. All sequences recorded separate sections through the lymph nodes.

### 2.3. Image Preprocessing

**Figure 1:**
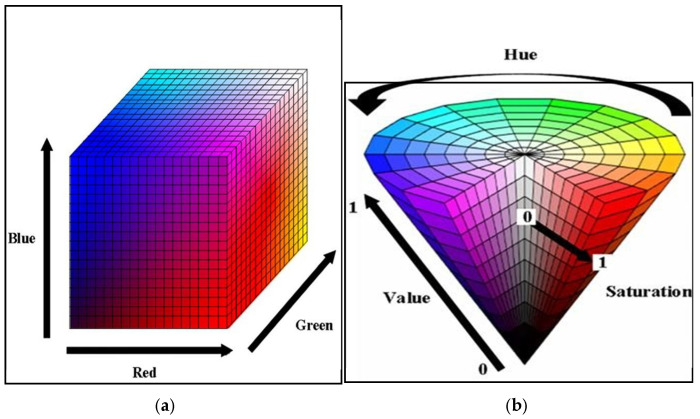
(**a**) RGB colour space and (**b**) HSV colour space [10].

Within the scope of this study, we examined the lymph nodes in MR slices using RadiAnt (in collaboration with radiologists) and manually marked the lymph node perimeter in green. We preprocessed these marked raw images in MATLAB (2023b software) to crop them automatically and generate the ground truth. Developing an algorithm capable of detecting closed green geometries and cropping them with their surroundings is vital for generating a reliable ground truth in manual lymph node segmentation. In order to create a mask in this study, which will involve using many datasets, the researchers implemented a new and effective preprocessing approach. For this purpose, the researchers converted the images from RGB (Red, Green, Blue) colour space to HSV (Hue, Saturation, Value) colour space in order to detect the green colour, which defines colours based on three basic properties [7]. As seen in Figure 1, the RGB space directly divides colours into red, green, and blue components, while the HSV space defines colours according to their hue, saturation, and brightness properties. By using this transformation, we can obtain a colour space that is more suitable for detecting objects with green tones. The complexity and inhomogeneity of the colour (chrominance) and intensity (luminance) information make RGB colour space not preferred for colour-based detection and analysis [8]. Automatic cropping operations particularly favour the transition to HSV space [9].

In HSV space, Hue refers to the position on the colour wheel. Hue varies circularly across the spectrum and is the basic characteristic of colours such as red, blue, green, and yellow. Normally, people express it as an angle between 0 and 360 degrees, but in its normalized form, it takes a value between 0 and 1. Hue values between 0.2 and 0.3 correspond to the green colour range, but for this study, the Hue range was determined as follows, since values between 0.2 and 0.4 may include the green colour range [11]. Saturation (saturation) refers to how pure or pale a colour is, taking a value between 0 and 100%. Here, 100% is a full and vivid pure colour, while 0 is a colour with more grey tones. Value determines how much brighter the colour is, i.e., how light or dark it is. Again, you can express it between 0 and 100%, but you can normalize it by determining a value between 0 and 1 for both Saturation and Value. In this study, we considered values between 0.3 and 1 for Saturation in order to avoid interfering with the grey colours in the MR image. We determined values between 0.1 and 1 to include all green colour brightness levels. We used the pseudocode of the algorithm presented in Algorithm 1.
**Algorithm 1:** Pseudocode of the algorithm for trimming the marked lymph nodes**1.*****img = imread(imagePath); ****\\ Read the image***2.*****hsvImg = rgb2hsv(img); ****\\ Convert from RGB to HSV***3.*****hueMin = 0.2; ****\\ Define the green colour range* ***hueMax = 0.4;*** ***saturationMin = 0.3;*** ***saturationMax = 1;*** ***valueMin = 0.1;*** ***valueMax = 1;*****4.*****mask = (hueMin <= hsvImg(:,:,1)) & (hsvImg(:,:,1) <= hueMax) & …******      (saturationMin <= hsvImg(:,:,2)) & (hsvImg(:,:,2) <= saturationMax) & … (valueMin <= hsvImg(:,:,3)) & (hsvImg(:,:,3) <= valueMax); ****\\ Find green pixels***5.***\\ Close the gaps between the green lines* ***se = strel(‘disc’, 1);*** ***mask_filled = imfill(mask, ‘holes’);*** ***mask_filled = imclose(mask_filled, se); ****\\ Closing operation***6.*****mask_filled = imfill(mask_filled, ‘holes’); ****\\ Merge the components***7.*****mask_filled = imerode(mask_filled, strel(‘disc’, 2)); ****\\ Clean the speckles***8.*****stats = regionprops(mask_filled, ‘BoundingBox’); ****\\ Find the count of green regions*
***greenRegionCount = length(stats);*****9.*****   for i = 1:greenRegionCount*** *\\ Start loop for each region****bbox = round(stats(i).BoundingBox);*****10.*****             if ~is_inside_cropped_regions(bbox, croppedRegions)*** *\\ If this region is not already cropped****                         if is_close_to_other_regions(bbox, croppedRegions, proximityThreshold) \\ If*** *region is close to other regions****                         continue; ****\\ Skip****end if****     \\ Expand the region****         expanded_bbox = expand_bbox(bbox, expansionBorder);***
*         \\ Save the cropped region****         save_image(cropped_region, [‘cropped_region_’, num2str(i), ‘jpg‘]);***
*\\ Add the cropped region to cropped regions list* ***croppedRegions [7] = expanded_bbox; end if******end for***

As we can see in Algorithm 1, we perform HSV transformation after reading the images. The algorithm detects green-coloured pixels between the maximum and minimum values. Then, we use morphological operators. First, we create a disc-shaped structural element called “se” and use the imfill function to close the gaps between the detected green lines. When the radiologist marks the lymph nodes, this process closes the gaps between the lines. Here, holes indicate that the gaps between the lines will be closed. The reason the imfill function is used twice in the algorithm is to eliminate the possibility that there may still be gaps between the lines despite the first operation. Afterwards, we use the imerode function to remove the thickening of the drawn area or the speckles that may occur after the filling process [12].

**Figure 2:**
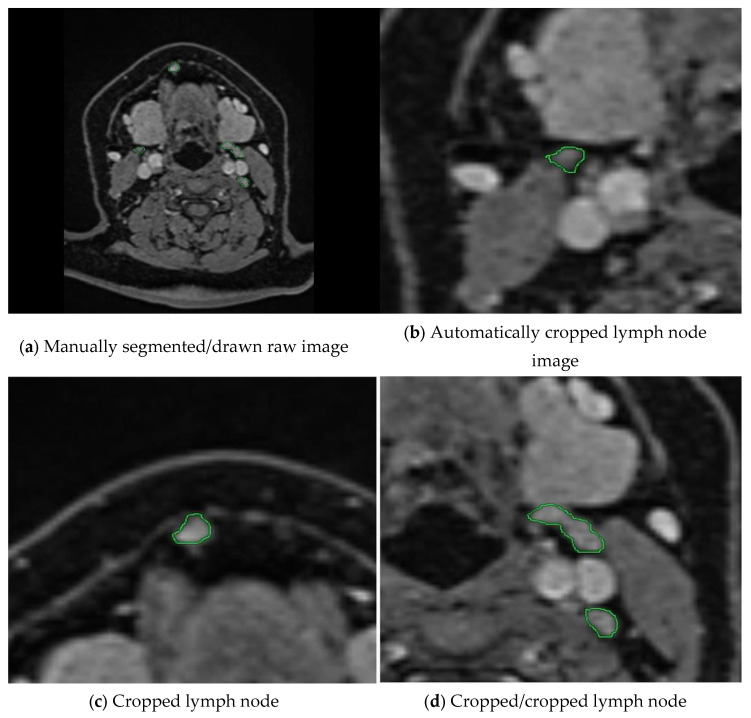
The workflow of our preprocessing program for semi-automatic segmentation is illustrated. (**a**) A raw medical image where lymph nodes have been manually annotated by an expert using green markings. (**b**–**d**) show the cropped images obtained by our proposed method, which automatically extracts the annotated regions. Our program crops each lymph node marked by the expert and ensures that the same lymph node is not cropped more than once through a control mechanism. This prevents redundant processing and ensures that each lymph node is included only once in the dataset.

In the next step, we calculate the rectangles called bboxes that surround the boundaries of closed geometric shapes in the binary images. After determining the number of these rectangles, i.e., the number of regions drawn with a green line, the loop is started. From this point onward, we verify two conditions. In this process, it expands the perimeter of the bbox by expanding the Expansion Border Value. In this study, we chose the threshold value as 150 pixels for the dataset we have. Thus, we perform cropping by expanding 300 pixels on each axis. The second condition is whether the currently controlled bbox is included in another bbox up to the proximity threshold value. If these regions are within 150 pixels of each other, which is the specified threshold value, this lymph node passes this region as cropped. If not, it will perform cropping at the expansion border value. This control mechanism, as shown in the example in Figure 2, controls the cropped areas and prevents repeated cropping of the same lymph node.

**Figure 3:**
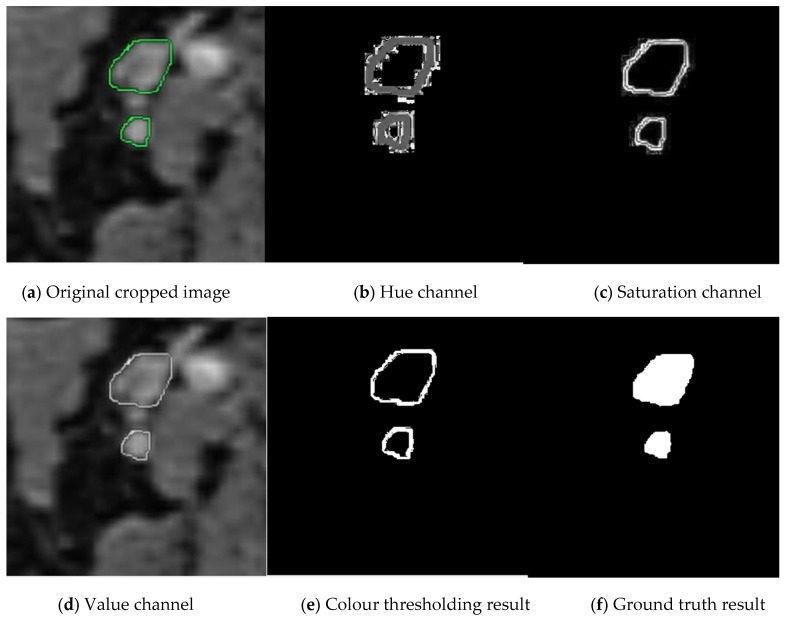
Stages of creating ground truth from cropped images. The manually cropped lymph node is marked in green, while the lymph node during ground truth creation is marked in shades of gray.

In the next stage, we convert the cropped images into binary images as masked images (ground truths). We apply the operations in Algorithm 1 with the same threshold values until the 7th row. Because the closed geometries drawn in green again need to be detected, their boundaries need to be determined, and the surrounding speckles need to be removed. We crop and save the resulting binary ground truths as the equivalent of each cropped image. In Figure 3, you can examine the original image and the image of each Hue, Saturation, and Value channel after switching to HSV space. You can observe images of the stages leading up to the ground truth by following the colour thresholding process and morphological operations.

**Figure 4:**
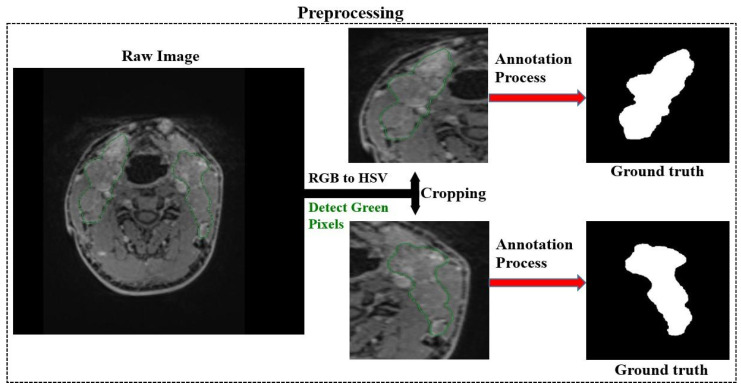
Automatic cropping of images and generation of ground truths.

Figure 4 summarises the preprocessing, which includes cropping the images and creating ground truths corresponding to the cropped images. The preprocessing mechanism automatically cropped the images in the same coordinates as the un-drawn and drawn versions of the cropped images.

These operations reduce the time spent while preparing the dataset. Although we perform these operations on the dataset we use and determine the parameters, we expect we can improve the method and develop new methods to reduce the time and effort required for dataset preparation. We hope that the number of publicly available datasets will increase with the spread of tools prepared in this way. After preprocessing, the experts automatically cropped the lymph nodes together with their surroundings and created ground truths by cropping the corresponding raw images in the same way. The experts performed these operations on all images in five MR sequences: DWI, T2, T1+C, T1, and ADC. The number of images after preprocessing is as in Table 1.

### 2.4. Study Design

**Figure 5:**
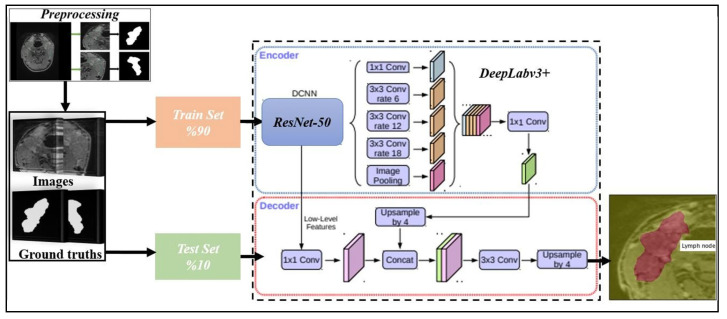
Proposed method.

After preprocessing, we obtained ground truths of the cropped lymph nodes with the MR images. We propose a model based on DeepLabv3+, one of the best segmentation networks, for the segmentation of lymph nodes. The DeepLabv3+ algorithm performs image segmentation using a coding–decoding structure [13]. As seen Figure 5, the coding part consists mainly of the neural network and the atrous spatial pyramid pooling (ASPP) module [13,14].The encoder part uses a pre-trained deep neural network model as a backbone network to extract high-level features from the input image [13,14]. Atrous (dilated) convolutions in the ASPP module improve segmentation performance by handling context information at different scales in parallel. This effectively integrates both broad and local context information [13,15]. The decoder is responsible for transforming the feature map produced by the encoder into a lower resolution output. The decoding part enlarges the output feature map and combines it with low-level features to obtain the final segmentation result [13,15]. The DeepLabv3+ algorithm combines high accuracy and fast processing capabilities to provide an effective solution for object segmentation.

**Figure 6:**
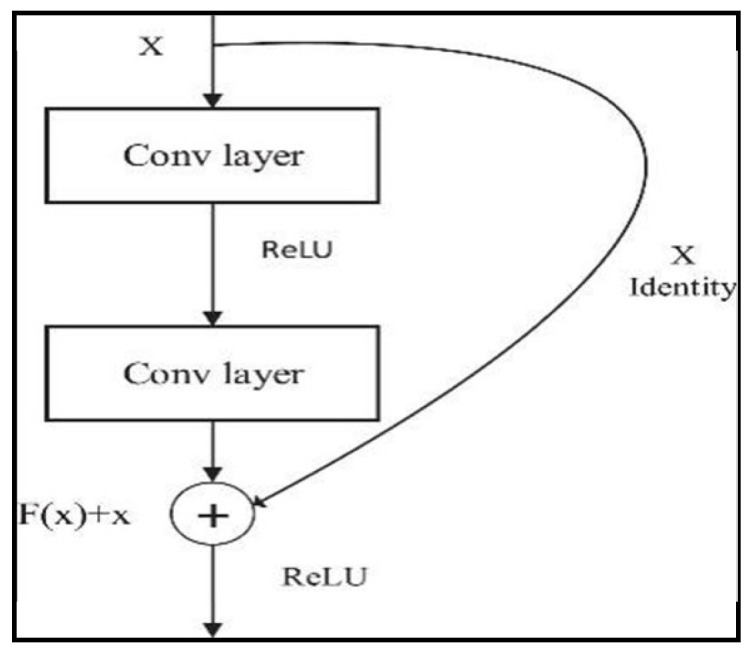
Residual block.

The encoder block of the DeepLabv3+ network uses the ResNet-50 network as its backbone network. This block not only increases the depth of the network but also improves the performance of the network [16,17]. ResNet-50 is widely used for image classification tasks and achieves brilliant success in detecting deep features. These hopping connections allow the gradient to flow from the input directly to the output and use a 50-layer structure to enable information transport in the network [16,18]. Each layer contains this structure, called a “redundant block”. Equation (1) expresses the mathematical expression for a redundant block in a ResNet-50:

*y* = (*x*, {*Wi*}) + *x* (1)

where *x* is the input of the block, *y* is the output of the block, {*Wi*} denotes the parameters of the block, and *F* represents a function including convolution layers, batch normalisation, and activation functions. Ref. [7] refers to the parameters of the block. These hopping connections facilitate training by allowing gradients to back-propagate more efficiently and allow the network to be deeper [17,19]. As can be seen in Figure 6, the residual block collects the input data and the output of the convolution layer to form a residual structure [17]. This structure allows the network to transmit the output of the previous layer directly to the next layer, while at the same time adding more information to this output.

Within the scope of this study, we used the Res-DeepLabv3+ model, which is based on the ResNet-50 network, for segmenting lymph nodes. We employed the proposed model to perform segmentation on all images of five MR sequences separately.

## 3. Results

In the context of this study, we trained and tested all images belonging to the DWI, T2, T1+C, T1, and ADC sequences with the proposed model. We divided 90% of the images in each sequence randomly for training and kept 10% for testing. We trained each sequence with the same training option settings as the Res-DeepLabv3+ model. Table 2 shows the training option parameters that were determined for training the Res-DeepLabv3+ model.

**Figure 7:**
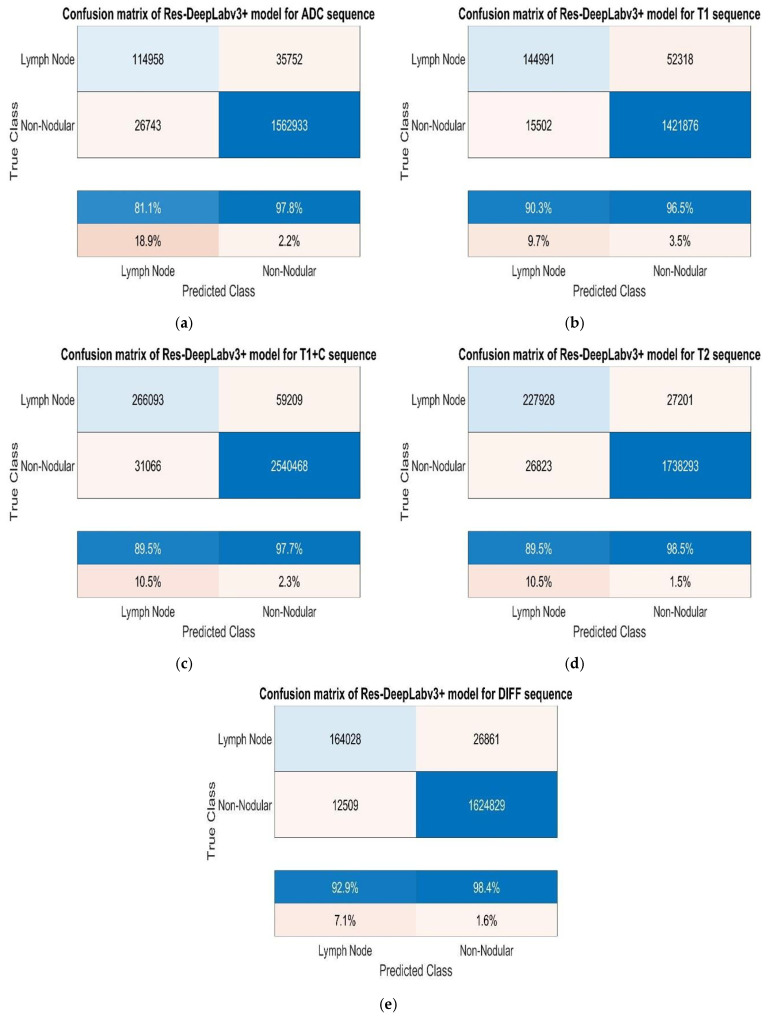
(**a**) Test confusion matrices without augmentation for ADC, (**b**) T1, (**c**) T1+C, (**d**) T2, and (**e**) DWI sequences.

As shown in Table 2, we performed 30 epochs of cervical lymph node training. Additionally, we set the mini batch size as 16 and the initial learning rate as 0.001. We performed the studies on a computer with NVIDIA GeForce GTX 1650 Ti, Intel (R) Core (TM) i5-10300H CPU @ 2.50 GHz, and 16 GB RAM. We used random rotation for augmentations and quadrupled the number of images. Figure 7 shows the confusion matrices obtained from the study conducted without augmentation.

**Figure 8:**
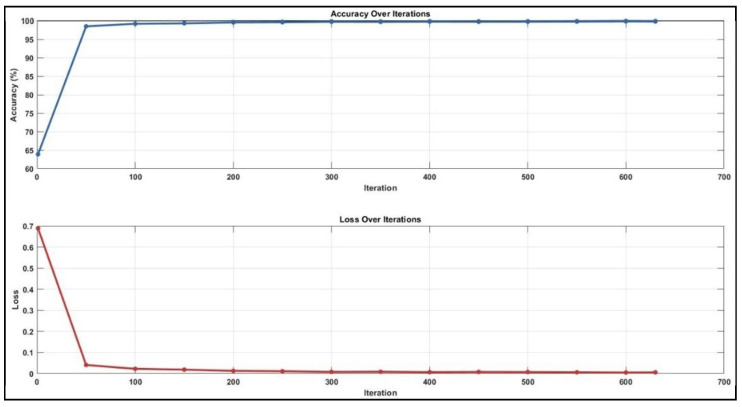
Training graph for DWI sequence with unenhanced dataset.

While analysing the confusion matrices in Figure 8, we observed that the ADC sequence showed the lowest results. By analysing the confusion matrices in Figure 8, it is evident that the DWI sequence produces the best results, even though all other sequences are also competitive. We obtained the true positive (TP), true negative (TN), false positive (FP), and false negative (FN) values for pixels classified as lymph node and non-nodular as 164,028, 1,624,829, 12,509, and 26,861 pixels, respectively. When normalised on a column basis, it correctly predicts the pixels of lymph nodes without augmentation at a high rate of 92.9%. Figure 8 shows the training graph for the DWI sequence for 30 epochs and 630 iterations. When analysing the graph, we observe that the training loss decreases as the accuracy increases.

**Figure 9:**
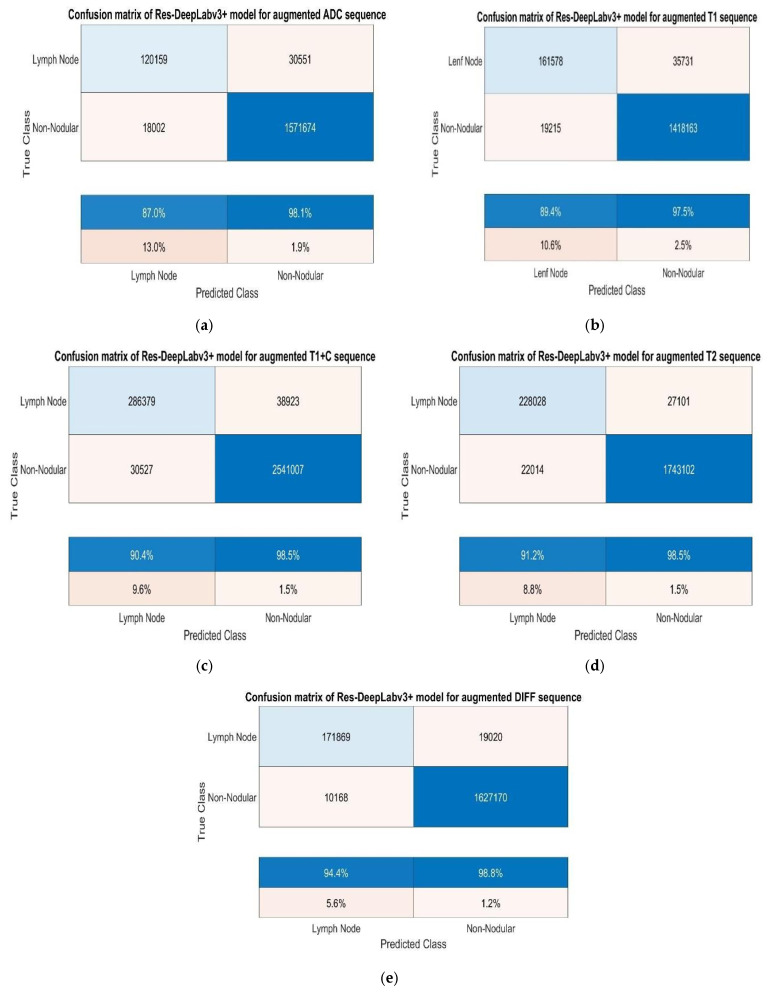
(**a**) Test confusion matrices after augmentation for ADC, (**b**) T1, (**c**) T1+C, (**d**) T2, and (**e**) DWI sequences.

After augmentation, we trained the Res-DeepLabv3+ model for all sequences using the same training parameters. Figure 9 shows the confusion matrices obtained for each sequence.

**Figure 10:**
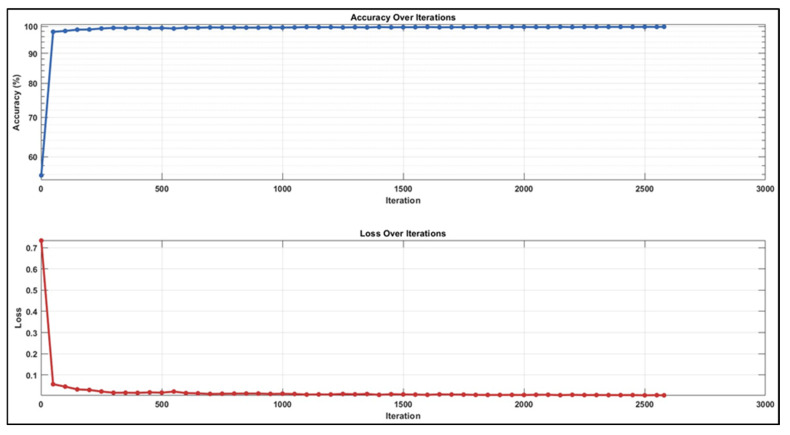
Training graph with augmented dataset for DWI sequence.

When we analysed the confusion matrices, we observed that all sequences have competitive confusion matrices, but the DWI sequence stands out. We obtained TP, TN, FP, and FN values for pixels classified as lymph node and non-nodular as 171,869, 1,627,170, 10,168, and 19,020 pixels, respectively. When normalizing the values in the confusion matrix by column, it correctly predicts the pixels belonging to lymph nodes at a very high rate of 94.4%. Figure 10 provides the training process graph for the DWI sequence that shows the best results.

According to the results of the validation set, as seen in Table 3 of the dataset without augmentation created for cervical lymph node segmentation, the mean IoU values for the DWI, T2, T1 T1+C, and ADC sequences are 0.89, 0.88, 0.81, 0.85, and 0.80, respectively. As seen in Table 4, the mean IoU values for all sequences in the augmented dataset are 0.91, 0.89, 0.89, 0.85, 0.88, and 0.84. 

## 4. Discussion

Currently, the gold standard diagnostic method of cervical lymph node pathologies is a histopathological examination after biopsy. Accurate preoperative diagnosis of cervical lymph node pathologies is a time-consuming process in the clinic. Imaging modalities occupy a very important place in the diagnosis of cervical lymph nodes. The diffusion sequence of MRI is the most effective sequence to distinguish cervical lymph nodes, even if they are smaller than one centimeter [20].

Medical activities widely use artificial intelligence, a new technology that simulates human intelligence. Although it is new, deep learning, which is used in every aspect of our lives, has also been involved in the diagnosis process of cervical lymph nodes. Various AI algorithms have emerged to detect lymph node pathology [21]. However, there are currently few studies that apply machine learning technology for the accurate diagnosis of lymph nodes [22].

Early diagnosis of head and neck cancers requires the recognition of suspicious lesions on radiological images. Seeing and marking the lesion on the radiologic image is called segmentation. Correct segmentation is very important for early diagnosis.Manual segmentation performed by radiologists is time consuming and the radiologists’ experience limits its accuracy. In recent years, automated segmentation has attracted great interest [23].

Deng et al. [23], in an automatic segmentation study using only 120 Dynamic contrast enhanced MR images obtained from a group of patients diagnosed with head and neck cancer, achieved a segmentation success of 86%. This study [23] achieved the highest value among the automatic segmentation studies in this field. In our study, we used five different sequences and over 200 MR images per sequence to segment cervical lymph nodes. In our study, the success rate of automatic segmentation using the T1 contrast-enhanced MR sequence was 89.5%. While Deng et al. [23] segmented head and neck masses in their study, we segmented reactive lymph nodes, lymphoma, and cervical lymph nodes with squamous cell carcinoma metastasis in our study. Our study is the first and only study in the literature which has used five different sequences, using deep learning architectures, for segmenting cervical lymph nodes in a comparative manner. Thus, our study will be a basic stepping stone for future studies in the classification of cervical lymph nodes generated by deep learning architectures on neck MRI images.

In some studies, researchers have shown that artificial intelligence can detect lymph node metastasis in breast cancer [24], lung cancer [25], and colorectal cancers [26]. Recently, there have been some radiomic studies to predict lymph node metastasis or extra nodal spread of head and neck squamous cell carcinoma from CT imaging. However, CT has the disadvantage of only being able to detect sufficiently large lymph nodes [27,28]. The MRI diffusion sequence is very successful in detecting small lymph nodes. Our study found that the model we developed had a higher success rate in detecting lymph nodes in the diffusion sequence compared to the other sequences.

In one study, the researchers stained lymph nodes excised from patients with head and neck squamous cell carcinoma using haematoxylin eosin stain, and they created two separate datasets with and without metastasis from the pathology preparations. By creating a CNN-based deep learning model, researchers have shown that this model can diagnose all positive lymph node images and exclude three quarters of negative lymph node images. To our knowledge, this is the first deep learning study that can detect metastasis from lymph node pathological images in head and neck squamous cell carcinoma [29]. As seen here, visual material is required to produce a study with artificial intelligence. This visual material may be a histopathological preparation or the MRI image in our study. In their study, Sünnetçi et al. [18,30] conducted two deep learning-based studies for parotid gland segmentation and the differential diagnosis of parotid gland pathologies. These studies observed that deep learning-based models were clinically successful and clinicians could use these systems to aid in early diagnosis. In this segmentation study, we achieved more success compared to previous studies. The higher number of patients and MR slices can explain the higher success rate in our study.

Nowadays, we use MRI to evaluate and manage nasopharyngeal carcinoma cases. MRI allows us to determine the size and margins of the mass in the nasopharynx. A study by Huang et al. compared the use of computer-assisted tumour segmentation methods to ease the workload of clinicians. Huang et al. used 253 MRI slices containing nasopharyngeal carcinoma lesions to evaluate the performance of the contour-based methods and the region-based method, and they showed the superiority of deep learning methods in both methods. This study is one of the rare studies that we encountered in the literature review which is orientated towards the mass and not the lymph node performed with MRI [31].

In a systematic review conducted in 2022, they analysed 13 studies that used artificial intelligence to classify cervical lymph nodes in the last 10 years. All the studies were retrospective and single-centre studies. Most of the studies investigated patients with oropharyngeal or oral cavity SCC. Fewer studies addressed laryngeal, nasopharyngeal or salivary gland SCC. The imaging modalities used in these studies were CT and PET/CT [32]. None of the identified studies have investigated the role of MRI for segmenting cervical lymph nodes using artificial intelligence. We aimed to segment lymph nodes with our deep learning model using MRI. The advantages of MRI are that magnetic resonance imaging does not contain radiation and reveals soft tissues better than CT, and MRI can recognise smaller lymph nodes compared to CT. The sentinel lymph node is the first lymph node to which a tumour drains and reflects the status of the entire lymphatic system. Using this procedure can reduce morbidity by avoiding unnecessary lymph node dissections and aids in accurate staging. Deep learning-based automatic segmentation methods, such as those presented in our study, facilitate the rapid and precise identification of sentinel lymph nodes. This advancement offers significant benefits in surgical planning and postoperative follow-up [33]. Moreover, these methods can help identify and distinguish artifacts resulting from various causes in postoperative imaging, thus minimizing false positive or false negative results and contributing to accurate patient diagnosis and treatment [34].

Evaluating the sections in MRI requires experience and expertise. In our study, we manually identified these lymph nodes with experienced head and neck radiologists and trained our deep learning model with these images. Because of our study, the ability of deep learning architectures to segment lymph nodes was parallel to the success of segmenting lymph nodes by an experienced radiologist. The reasons for fewer studies with MRI may be the long MRI acquisition time, not being performed frequently in routine clinical practice, and motion artifacts distorting the MR image. In our study, we excluded patients with motion artifacts.

The number of sections obtained using MRI in our study is the highest number of sections compared to previous studies. In our study, according to the test set results, the mean IoU values for the DAG-b1000, T2, T1, T1+C, T1+C, and ADC sequences in the dataset without augmentation created for cervical lymph node segmentation are 0.89, 0.88, 0.81, 0.85, and 0.80, respectively. In the augmented dataset, the average IoU values for all sequences are 0.91, 0.89, 0.85, 0.88, and 0.84.

In our study, augmentation did not significantly change the success rate since the number of patients and sections was sufficient. The large number of sections in our study enabled the recognition of lymph nodes in different parts of the neck and increased the predictive ability of our model.

In part of our study, we used automatic segmentation to eliminate the necessity of manually reprocessing the selected lymph nodes. Automatic segmentation prevented the loss of time in manual segmentation studies. Mahdavi et al. [35] showed in their study on semi-automated segmentation in prostate interventions that the proposed semi-automated segmentation method outperformed the manual segmentation method in terms of speed and had many advantages.

Manual image segmentation, which is routinely performed in radiotherapy to identify the anatomical structures of each patient, is a time-consuming procedure. In radiation oncology, correct segmentation is crucial for the effectiveness of the radiotherapy plan, because the treatment plan is based on the creation of these zones. Treatment success depends on this plan. Automatic segmentation eliminates the differences in the experience of physicians performing manual segmentation, minimizes the details that may be overlooked in manual segmentation, and introduces a standard approach [36].

Developing a more accessible programme using the preprocessing technique created within the scope of this study will enable us to create additional datasets for segmentation studies on MR images faster.

In our study, the fact that we could compare the findings by using five different MRI sequences made up its strength and difference from the literature. Our study showed that the most successful sequence in MR is the diffusion sequence to segment smaller lymph nodes. The limitations of our study are that we conducted a retrospective study, had few patients, used a single centre, did not examine the levels of lymph nodes, and did not take an equal number of images from each sequence.

## 5. Conclusions

The evaluation of MR images taken to detect cervical lymph node pathologies requires experience and expertise in radiology. This is because the neck has a complex anatomical structure and the lymph nodes are quite small and do not have a fixed position and shape. In this study, the model we created with the deep learning architecture was able to segment cervical lymph nodes with high accuracy in MR slices. Just like an experienced radiologist, it could mark the cervical lymph node regardless of whether it was pathological. This success was also shown in five different MR sequences. The diffusion sequence achieved the highest success rate in detecting lymph nodes smaller than 1 cm compared to any other sequence.

This achievement allows for the rapid and highly accurate recognition of cervical lymph nodes. It speeds up the diagnosis and treatment process. It allows physicians who have little experience with neck MRI to recognise cervical lymph nodes in a short time.

This study makes up the cornerstone of a huge pyramid. In this study, we have created a model that can mark cervical lymph nodes quickly and with high accuracy, regardless of whether they contain pathology, using MR images. With the increase in the number of patients and diagnostic diversity and developments in deep learning architectures, future studies may lead to changes in the algorithms in the diagnosis of neck masses. This study may contribute to the creation of new models that can predict the benign–malignant differentiation of lymph nodes with high accuracy before surgical procedures, radiotherapy, or chemotherapy. The automatic segmentation algorithm used in this study may replace manual segmentation in radiotherapy, thus increasing the effectiveness of radiotherapy. These models will guide diagnosis and treatment processes.

## Figures and Tables

**Table 1:** Number of images before and after preprocessing.

MR Sequence	Number of Raw Images	Number of Cropped Images After Preprocessing
DWI	256	382
T2	259	377
T1+C	380	536
T1	213	305
ADC	236	363

**Table 2:** Training parameters for the Res-DeepLabv3+ model.

Training Parameter	Value
Momentum	0.9
Initial Learning Rate	0.001
Epochs	30
Learn Rate Drop Factor	0.1
Learn Rate Drop Period	10
Mini Batch Size	16
L2 Regularisation	0.0001
Gradient Threshold Method	l2norm
Validation Frequency	50

**Table 3:** Results without augmentation.

MR Sequence	Global Accuracy	Mean Accuracy	Mean IoU	Weighted IoU	Mean BF Score
DWI	0.97847	0.92582	0.89139	0.9586	0.85383
T2	0.97326	0.93909	0.88913	0.94947	0.82222
T1+C	0.96884	0.90295	0.85618	0.94109	0.76852
T1	0.95851	0.86203	0.81789	0.9215	0.69665
ADC	0.96409	0.87298	0.80469	0.93438	0.72259

**Table 4:** Results with x4 augmentation.

MR Sequence	Global Accuracy	Mean Accuracy	Mean IoU	Weighted IoU	Mean BF Score
DWI	0.98403	0.94708	0.9186	0.96906	0.88787
T2	0.97569	0.94065	0.89769	0.95368	0.84069
T1+C	0.97603	0.93424	0.88911	0.95447	0.81648
T1	0.96639	0.90277	0.85447	0.93657	0.75552
ADC	0.9721	0.89298	0.84112	0.94771	0.77289

## Data Availability

The original contributions presented in this study are included in the article; further inquiries can be directed to the corresponding author.
